# Engineered Nanoparticles for μPAD Nucleic Acid Detection

**DOI:** 10.1021/acsomega.4c11390

**Published:** 2025-05-13

**Authors:** R. Luc Morgan, Lillian J. Zehnder, Madeline A. Jenkin, Erika Alonso, Sasha Haunz, Abby Bevil, Daniel F. Scott

**Affiliations:** Department of Chemistry, 2779 Centre College, Danville, Kentucky 40422, United States

## Abstract

Point-of-care (POC) diagnostics can provide early disease detection and continued monitoring for millions of people, domestically and internationally, who do not have access to essential support services. POC diagnostics can improve care and treatment decisions by shortening the time from analysis to diagnosis. Current POC diagnostic systems are limited by the availability of analyte options, nonspecific responses, the need for trained personnel, specialized instrumentation, and expensive biological components. Herein is presented a novel approach to develop POC diagnostics based on iron oxide@gold, core@shell, nanoparticles (Fe_3_O_4_@Au’s). The particles were engineered to release signaling compounds in the presence of the target analytes and built on a paper-based microfluidic (μPAD) platform to allow for inexpensive production and high portability. As a proof-of-concept, an assay for the oligonucleotide target sequence of survivin was developed. The nanoparticles were designed to release a signal in the presence of survivin DNA, which was quantified via a fluorescence signal on the μPAD platforms. The system showed selective response for the targeted survivin sequence, and detection was achieved with buffer, artificial saliva, artificial urine, and human serum matrices.

## Graphical abstract

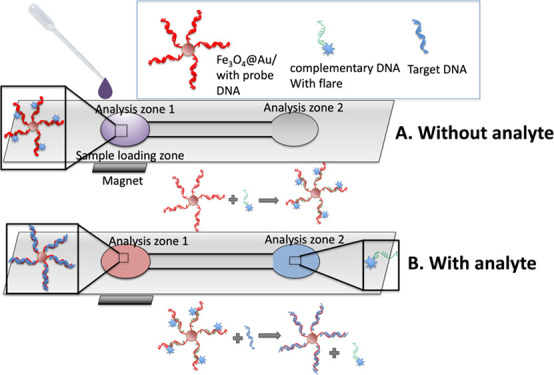

## Introduction

Point-of-care (POC) diagnostics have dramatically changed the medical landscape by offering access to quick, portable, and simple diagnostic and analytical feedback. One early example is a dipstick formulated for glucose quantification from the 1950s.^1^ Since then, POC devices have become a mainstay for diagnostic detection, including glucose monitors for diabetics,^2^ at-home pregnancy tests,^3^ and at-home COVID-19 tests.^4^ Beyond convenience, POC systems can greatly improve the prospects for lower socioeconomic communities and low- and middle-income countries (LMICs). While early diagnosis and continued monitoring are critical to improve outcomes for both communicable and noncommunicable diseases, access to care is limited for individuals in particular geographic locations. Many LMICs lack critical pathology services, worsening the disease impact in these countries.^5^ Availability of POC diagnostic devices for use at home or in clinical settings in remote areas can potentially encourage individuals to seek medical care and provide information for rapid initial diagnosis and disease management. As exemplified by the COVID-19 pandemic, rapid analysis can slow the spread of a communicable disease by identifying infected individuals.

Ideally, a POC device is simple to use, rapid, accurate, and inexpensive to manufacture. They can be constructed from a diverse range of materials and components, enabling them to be specialized for a variety of applications. POC devices have been developed on multiple detection platforms, including lateral flow immunoassays (LFIA),^6,7^ lab-on-a-chip and lab-on-a-disc microfluidics,^8,9^ and paper-based microfluidic devices (μPADs),^10^ with each platform having advantages and disadvantages. While paper-based analytical tests can be traced back to litmus paper in the 1700s,^11^ μPADs are a relatively recent addition to the POC platforms, with the first example coming from the Whitesides group using photolithography patterning in 2007.^12^ The premise of μPAD technology is to pattern hydrophobic barriers to direct flow and create microliter volume channels on a porous membrane (Figure 1).^13^ Using different patterning techniques allows unique flow patterns beyond the straight lines seen with nitrocellulose membranes used in many of the lateral flow assays.^14–16^ The differences in patterns make multiplexed analysis and sample pretreatment possible. Patterning has been achieved by a variety of techniques, including photolithography,^14^ wax printing,^15^ wax dipping,^17^ inkjet printing,^16^ chemical vapor deposition,^18^ stamping,^19^ and cutting.^20^ Of the available options, printing offers the convenience of using standard software (Microsoft Word, PowerPoint, etc.) to design the hydrophobic pattern. With the printing methods in particular, once the design has been printed, the paper (typically a type of chromatography paper) can be heated to allow the wax/ink to permeate the paper and create the hydrophobic barriers and microfluidic channels.^21^

**Figure 1:**
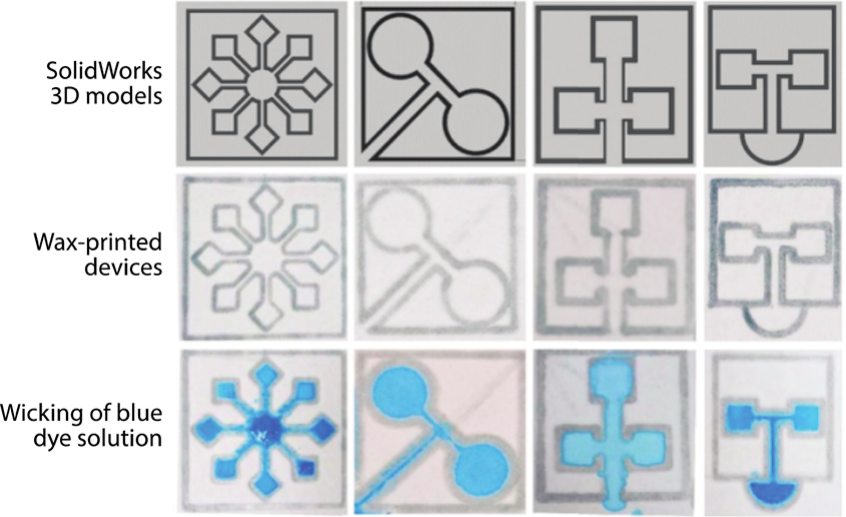
Example of a hydrophobic barrier creating microchannels, patterned by a wax printer. Figure from ref 13.

Beyond choices for the platform and patterning configuration, options for the signal transduction and readout mechanism of POC devices can include a variety of different mechanisms. Electrochemical readouts are popular in POC devices,^22^ with applications based on voltammetric,^23,24^ impedimetric,^25–27^ and potentiometric^28–30^ techniques. Electrochemical readouts offer high sensitivity and fast response time but suffer in terms of stability, interference, and the requirement of electroactive species. Fluorescence POC detection offers high sensitivity with low cost and rapid analysis and has been performed using both fluorescent molecules^31,32^ and nanomaterials.^33,34^ Fluorescence can be generated^35,36^ or quenched,^37,38^ depending on the system, but requires labeling the analyte with a fluorophore. Additionally, fluorescence instrumentation is required and can be susceptible to high background fluorescence and photobleaching. Surface-enhanced Raman scattering^39,40^ and surface plasmon resonance^41,42^ both offer improvements in sensitivity and selectivity with low background signals but require bulky and expensive equipment which can limit the portability and the analysis time. Magnetic readout has garnered attention for demonstrating high specificity and sensitivity while lacking the need for optical components and having minimal background.^43,44^ Magnetic detection devices, however, can be time-consuming and expensive and require specialized instrumentation that is not readily available. Colorimetric readout offers simple and easy to visualize detection without instrumentation but can suffer from low sensitivity, lacks quantitative ability, and requires a detectable color change.^45–47^

Magnetic particles have shown utility in POC devices. Iron oxide nanoparticles are most often used for analyte enrichment and separation from the sample matrix, but have also been used for signal amplification and as the readout mechanism.^44^ When gold or iron oxide nanoparticles are used for readout or signal amplification, they are typically labeled with antibodies for analyte recognition. To take advantage of the surface chemistry and physical properties of gold and the magnetic ability of iron oxide, the two have been combined in Fe_3_O_4_@Au (core@shell) nanoparticles for sensors, including POC applications. Electrochemical detection of heavy metals has been achieved using DNA-modified Fe_3_O_4_@Au,^48^ while Fe_3_O_4_@Au SERS-based detection has been demonstrated for pathogenic bacteria^49^ and in a POC lateral flow assay for the analytes serum amyloid A and C-reactive protein.^50^ Currently, many available POC devices achieve analyte recognition by means of antibody binding, which can pose challenges. Antibodies are expensive to produce and are likely to be unstable under the storage conditions prevalent for field POC applications in remote locations.^51,52^ For many analytes, multiple antibodies are required to recognize the analyte and elicit the signal in the lateral flow format.^44^

Despite the growth in the POC field, there are still opportunities to improve the detection options and available analytes, as well as to tailor the devices for use by untrained personnel, with the potential to help millions of people worldwide.^5,53–58^ Compared with traditional LFA antibody detection, nucleotides are even more difficult to detect with many POC devices. LFA POC tests are typically performed on nitrocellulose membranes, in combination with a sample conjugation pad to introduce the sample to the assay components and an absorbent pad to encourage flow of the solution down the device through capillary action.^59^

To expand available mechanisms for POC device construction, we have developed an alternative approach for analyte recognition and signal generation using DNA-labeled Fe_3_O_4_@Au nanoparticles (Fe_3_O_4_@Au’s). As a proof-of-concept, the Fe_3_O_4_@Au’s were designed to respond to survivin DNA and release a signal molecule for detection. In general, cell-free DNA has been found circulating through biological fluids (urine, serum, saliva, etc.) and has been correlated to disease presence and/or activity.^60^ Survivin regulates cell division and inhibits apoptosis, displaying overexpression in most human cancers.^61,62^ Urine survivin has been used to diagnose bladder cancer,^63^ and high levels of survivin in serum are indicative of various types of cancer.^64^ The response was visualized by applying the sample to a wax-printed cellulose chromatography paper μPAD coupled with magnetic attraction for separation of the released from nonreleased signal molecules. To our knowledge, Fe_3_O_4_@Au nanoparticles have not been implemented as a platform for analyte recognition and signal production with the proposed mechanism described herein or used in combination with μPAD technology to make POC devices.

## Experimental Section

### Materials and Instrumentation

Unless otherwise stated, all chemicals, including the Fe_3_O_4_ nanoparticles, were purchased from Sigma-Aldrich (USA). DNA was purchased from and modified by Integrative DNA Technologies (Coralville, Iowa, USA). Fluorescence measurements were made on a POLARstar Omega Plate Reader (BMG LABTECH, Cary, NC, USA). Paper-based devices were printed with a Xerox ColorQube 8570 printer and Xerox wax. Cytiva Whatman 3MM Chromatography Paper was purchased from Fisher Scientific (Waltham, MA, USA). N52 strong neodymium magnets were purchased from Applied Magnets Superstore (Plano, TX, USA).

### Fabrication and Characterization of μPAD Platforms

All μPAD platforms were printed with a Xerox ColorQube 8570 printer on a Whatman 3MM chromatography paper. All templates were designed with Microsoft PowerPoint. After printing, the devices were baked for 4 min at 95 °C to create the hydrophobic barriers and seal the devices, confirmed by the addition of water to the microfluidic channel. After cooling, combinations of Fe_3_O_4_@Au’s, Cy3-tagged DNA, and Al647 (similar λ_ex_ and λ_em_ as Cy5) tagged DNA were added to the devices to assess the practicality of the different components with the nanoparticle system.

### Preparation of Fe_3_O_4_@Au Nanoparticles

The Fe_3_O_4_@Au nanoparticles were synthesized from an adapted procedure.^65^ Briefly, Fe_3_O_4_ superparamagnetic nanoparticles were diluted, mixed with 0.27 M EDTA in 1.0 M NaOH, and sonicated. The nanoparticles were then purified via centrifugation and washed. The Au shell was deposited by resuspending the Fe_3_O_4_ in cetyltrimethylammonium bromide (CTAB), followed by the addition of HAuCl_4_ in a basic solution. The solution was stirred vigorously before the addition of hydroxylamine hydrochloride and was continually stirred. The Au coating was allowed to deposit 24 h at room temperature, and particles were purified by centrifugation at 15,000 rpm for 15 min, with two wash steps before use. Fe_3_O_4_@Au nanoparticles were characterized by UV/vis spectroscopy.^66^

### DNA Functionalization of Fe_3_O_4_@Au and Au Nanoparticles

Single-stranded DNA sequences corresponding to the capture sequence and the complementary flare sequence were purchased individually (Table 1). The two strands were annealed together prior to the attachment to the Fe_3_O_4_@Au surface by combining in TE8 (10 mM TrisHCl, 1 mM EDTA, pH 8) buffer, heating to 95 °C, and cooling to room temperature. The capture sequence was attached directly to the Au surface with the addition of thiol functionality on the 5′ end to allow formation of the Au–S bond on the nanoparticle surface. The annealed DNA strands were incubated with excess Tris­(2-carboxyethyl)­phosphine hydrochloride (TCEP) to reduce the disulfide bonds formed between thiols on the individual DNA strands. The reduced double-stranded DNA was then added in excess to the Fe_3_O_4_@Au’s for 1 h at room temperature. DNA loading was aided by salt aging of the Fe_3_O_4_@Au/DNA solutions by increasing the [NaCl] to 50, 150, and 300 mM each hour after the initial incubation and stored at 4 °C overnight. Excess DNA not on the Fe_3_O_4_@Au’s surface was removed by repeated centrifugation and washing with TE8 buffer before final reconstitution in TE8 buffer. All solutions were stored at 4 °C until use.

**Table 1:** Different DNA Sequences Used in Combination with the Fe_3_O_4_@Au Nanoparticles to Detect the Presence of Survivin Target DNA

DNA strand	sequence	function
capture thiol	5′-CCC AGC CTT CCA GCT CCT TG-(A)_5_-propylthiol-3′	coat the nanoparticle surface and recognize the survivin target sequence
flare	5′ (Cy5)-TCA AGG AGC TGG 3′	anneal to the capture thiol sequence and anchor the flare
Survivin target	5′ CAA GGA GCT GGA AGG CTG GG 3′	target sequence for detection
three-base mismatch	5′-TCT AGC AGC TCG 3′	mismatched target sequence for specificity; mismatches are underlined
one-base mismatch	5′-TCA AGC AGC TGG 3′	mismatched target sequence for specificity; mismatch is underlined

### Detection of Target DNA

Fluorescence spectroscopy was used to provide initial data to confirm the effectiveness of the nanoparticle-DNA decoration and subsequent target-oligonucleotide-induced release of the flare sequence. The fluorescence from the Cy5-tagged flare sequence was largely quenched when annealed to the thiolated capture strand, which positioned the fluorophore near the surface of the nanoparticles. The reduced emission from Cy5 indicated the presence of DNA on the nanoparticle surface. The DNA-decorated nanoparticles were then incubated with the target oligonucleotides at differing concentrations. As the flare sequence was replaced by the target sequence, Cy5 moved away from the surface of the nanoparticle, and the full fluorescence emission was restored. Target survivin DNA standards were prepared at varying concentrations in TE8 buffer, artificial urine, artificial saliva, and human serum. Similar standards were prepared with the one- and three-base mismatched targets to assess the selectivity of the detection system. Appropriate blanks were prepared in the respective matrices, as well. The different concentration DNA standards in the respective matrices were mixed with detection Fe_3_O_4_@Au/DNA nanoparticles and incubated 15 min. The solutions were then added in triplicate to a 96-well plate, and the fluorescence intensity was measured with the Cy5 fluorescence filters (λ_ex_ = 620 nm, λ_em_ = 670 nm).

Once the flare sequences were fluorescently confirmed to be released from the surface of the nanoparticles by the target oligonucleotides, the nanoparticle detection system was translated to the μPAD paper-based platform. Many of the experiments from the fluorescence confirmation were mimicked, as the detection system was translated to the μPAD platform. The general schematic for the detection is shown in Figure 2. The Fe_3_O_4_@Au nanoparticles labeled with the capture and flare sequences, as described above, were incubated with varying target oligonucleotide concentrations in different matrices. After incubation with the target sequence samples, 40 μL of the mixture was added to the μPAD platform with the magnet secured under the loading zone area (analysis zone 1) with a micropipette, in triplicate. The solution was allowed to wick down the device while the loading zone was held over the magnet. The released flare that migrated to analysis zone 2 was quantified with fluorescence. The μPADs were secured on a 96-well plate, with the location of the analysis zone 2 corresponding to a specific well on the plate to be measured with the plate reader. The fluorescence intensity in analysis zone 2 was measured with the Cy5 fluorescence filters (λ_ex_ = 620 nm, λ_em_ = 670 nm). Data was plotted and normalized with GraphPad Prism 10.

**Figure 2:**
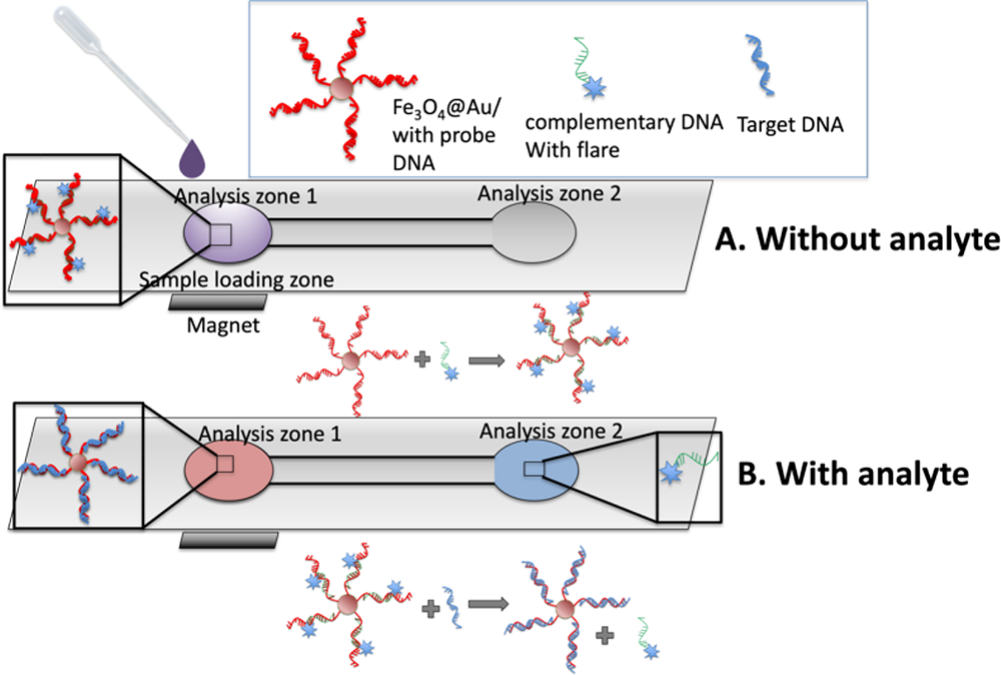
General schematic of the proposed μPAD. (A) In the absence of analyte, a single spot will be visible in analysis zone 1. (B) In the presence of the analyte, the flare will be release from the surface of the Fe_3_O_4_@Au and travel to analysis zone 2.

## Results and Discussion

Significant progress has been made in POC diagnostics, with advancements in technology leading to the development of more accurate, rapid, and user-friendly testing devices. However, there are still opportunities to expand the available disease detection options, reduce costs, and improve ease of use by untrained personnel to increase accessibility and affordability of these diagnostics and ensure equitable healthcare for all.^5,53–58^ To that end, we have developed a detection mechanism for inclusion in a POC device using Fe_3_O_4_@Au’s, which were initially combined with a wax-printed, μPAD platform. Our goal of this work was to create a portable and inexpensive detection mechanism for inclusion in assays to eventually allow on-site analysis by untrained personnel. We envision that the devices will find relevance in situations where advanced instrumentation and lab analysis are not possible. The preliminary device platform was patterned with a wax printer on cellulose chromatography paper to create the μPAD. In the future, this method will allow the pattern to be modified rapidly to find the ideal design and ultimately enable low-cost manufacturing. A full 8.5 in x 11 in sheet of paper containing more than 50 devices costs roughly $0.60 to produce, keeping the cost of each platform device to roughly one cent.^15^

Gold-coated iron oxide nanoparticles (Fe_3_O_4_@Au) were chosen as the basis of the analysis because of the desirable properties that each material possesses individually and the ability to be combined. Gold nanoparticles (AuNPs) have been widely used in POC devices because of their straightforward synthesis, visualization, bioconjugation with both thiols and amines, and biocompatibility.^67–70^ AuNPs are commonly used as optical readout components due to their deep color and high contrast. The surface of Au is easily functionalized by forming strong Au–S bonds with free thiols in solution.^71^ A Au–S bond is an ideal choice for nanoparticle conjugation to biological molecules, as the thiol chemical group can be added to oligonucleotide sequences^72^ or included in peptide sequences by incorporation of cysteine.^73^ Iron oxide nanoparticles possess several useful features and have also frequently been used in POC devices as components for visualization and magnetic separation.^44,74^ A feature exclusive to iron oxide nanoparticles is the superparamagnetic property that allows them to be strongly attracted to a magnetic field.^44^ By using an Fe_3_O_4_@Au combination system, the surface of the nanoparticle will be easier to functionalize while superparamagnetic properties are incorporated into the nanoparticles. While gold can be an expensive material, the use of the core@shell structure dramatically reduces the amount of gold needed and minimizes costs. In addition, combining the Fe_3_O_4_@Au’s with μPADs and microliter sample volumes decreases the amount of reagents needed for each test. With the presented diagnostic system, each test costs roughly $0.26 total in materials to produce.

μPADs served as the device platform and were the sample holder as well as the background for the readout. The light colored background provided high contrast and allowed sensitive detection.^45^ The remainder of the assay components relied on the Fe_3_O_4_@Au system for the analyte recognition and quantification. The surface of Fe_3_O_4_@Au was decorated with a “flare” molecule conjugated to the Fe_3_O_4_@Au surface via an analyte-sensitive linkage. After the sample solution was combined with flare-loaded Fe_3_O_4_@Au, the solution was added to the μPad platform. As demonstrated in Figure 2, the flare was initially attached to the surface of the Fe_3_O_4_@Au via noncovalent interactions with the probe. The strength of the noncovalent interaction between the probe and the flare was designed to be strong enough to keep the flare attached to the surface of the Fe_3_O_4_@Au’s in the absence of the target sequence. A magnet was positioned under the loading zone to secure the Fe_3_O_4_@Au’s and anything attached to the surface. If the target analyte was not present, the flare stayed attached to the surface of the Fe_3_O_4_@Au and the magnet restricted the Fe_3_O_4_@Au’s and the flare to analysis zone 1 (Figure 2A). However, in the presence of the target sequence, the interaction of the flare with the probe on the surface of Fe_3_O_4_@Au was replaced by the target sequence, triggering the release of the flare from the Fe_3_O_4_@Au. The flare was no longer confined to analysis zone 1 by the magnet and traveled through the microfluidic channel to analysis zone 2 (Figure 2B), where the signal was measured. Fluorescence readout was investigated initially because of its ease of interpretation, and quantifiable optical results were the ultimate goal, but additional readout mechanisms, including color intensity, alternative fluorescence mechanisms, and/or electrochemical readout could be investigated to improve the ease of use, cost, and/or analytical figures of merit (sensitivity, range, etc.) as needed.

### Fabrication and Characterization of μPAD Platforms

Wax patterning on a cellulose chromatography paper was chosen as the initial platform to build the devices. The initial designs were prepared using Microsoft Word and Microsoft PowerPoint and printed with a Xerox Colorqube 8570 printer on Whatman 3MM chromatography paper to confirm that hydrophobic barriers could be constructed to define microfluidic channels and allow the flow of solution. The devices were wrapped in aluminum foil and baked to allow the wax to permeate the paper. After cooling, combinations of Fe_3_O_4_@Au’s, Cy3-tagged DNA, and Al647 (same λ_ex_ = 620 nm, λ_em_ = 670 nm as Cy5) tagged DNA were added to the devices to assess the practicality of the different components with the magnetic system. Figure 3a shows that the flow of aqueous solutions was confined to the wax-printed channels, while Figure 3b shows the migration of Fe_3_O_4_@Au through the channel in the absence of a magnet. Once a magnet was placed under the circle portion of the top of the device, the flow of Fe_3_O_4_@Au was restricted (Figure 3c). Combinations of Fe_3_O_4_@Au with free Cy3- and Al647-tagged DNA were added to the analysis zone 1. As shown in Figure 3d,e, the magnet impeded the flow of the Fe_3_O_4_@Au’s to analysis zone 2 but allowed the Cy3 and Al647 tagged DNA to flow freely. The dark circles in the top portion of 3d and 3e are the Fe_3_O_4_@Au’s that were restricted to the initial loading zone. Together, these results demonstrate that the presence of color/signal in analysis zone 2 would indicate the presence of a target analyte, as the release of the fluorophore-tagged DNA from the Fe_3_O_4_@Au is dependent on that analyte.

**Figure 3:**
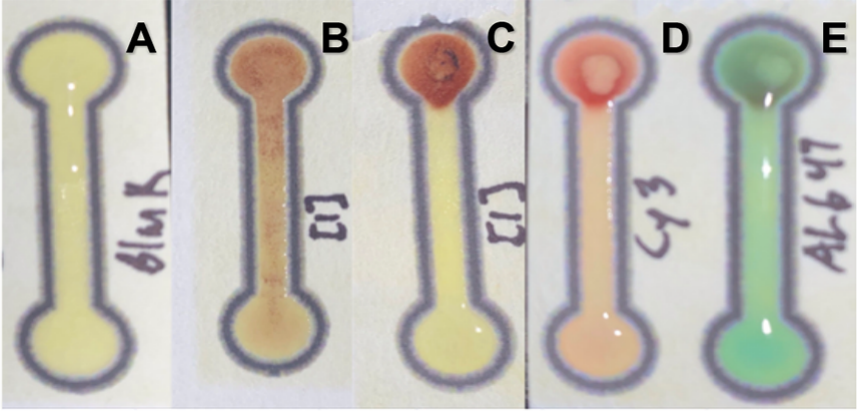
Performance of μPAD wax printed devices with different solutions. (A) TE8 buffer, (B) Fe_3_O_4_@Au and no magnet, (C) Fe_3_O_4_@Au and a magnet, (D) Cy3 tagged DNA with a magnet, and (E) Alexafluor 647 tagged DNA with a magnet. For any examples with a magnet, the magnet was placed under the top circle portion of the design.

### Preparation of Fe_3_O_4_@Au Nanoparticles

Fe_3_O_4_@Au nanoparticles were prepared starting with 20 nm Fe_3_O_4_ magnetic nanoparticles purchased and coated with gold following a previous procedure.^65^ After production of the Fe_3_O_4_@Au’s, the size and concentration were determined by UV/vis spectroscopy.^66^ Based on the UV/vis spectrum, the Fe_3_O_4_@Au’s were determined to be 36 nm in diameter and at a concentration of 1.85 × 10^–9^ M. Fe_3_O_4_@Au’s were diluted to 5.0 × 10^–10^ M before use.

### Detection of Target DNA

To demonstrate a proof of concept for a nucleotide target sequence, survivin DNA was chosen as a model analyte. The detection of the cancer target sequence survivin holds significant diagnostic importance due to its critical roles in cell division regulation and apoptosis inhibition, especially if it is found in the cell-free circulating form. Survivin is often overexpressed in a wide range of human cancers, making it a valuable indicator of malignancy.^61,62^ Its presence in bodily fluids such as urine and serum offers noninvasive diagnostic potential. For instance, urinary survivin levels are utilized in diagnosing bladder cancer, providing a simple and effective screening method. Similarly, elevated serum survivin levels are associated with various cancer types, aiding in early detection and diagnosis.^63,64^ For the survivin nucleotide target sequence (Figure 4a), the probe is a single-stranded DNA sequence complementary to the survivin sequence that is attached to the surface of the Fe_3_O_4_@Au surface through a Au–S bond. The flare is bound to the Fe_3_O_4_@Au through noncovalent attachment (hybridization) to a complementary single-stranded DNA sequence, with which the probe could hybridize. The probe DNA sequence was designed to be more highly complementary to the survivin target DNA in comparison to the flare. Thus, in the presence of the target DNA, the flare DNA will be replaced on the Fe_3_O_4_@Au surface and be free to migrate to analysis zone 2 (Figure 2). Prior to attempting the analysis of biomarker analytes on the chip, the release of the flare molecule from the surface of the Fe_3_O_4_@Au by the biomarkers was confirmed using fluorescence quenching technology. The system was designed to place the flare molecule in close proximity to the Au surface of the nanoparticle, which has been shown to quench fluorescence emission.^75^ Upon the interaction of the target sequence with the biomarker-specific probe on the Fe_3_O_4_@Au surface, the flare was released and fluorescence emission restored (Figure 4A schematic and Figure 4B results). The solution-based analysis enabled detection of 5.0 nM, or 31 ng/mL, survivin DNA. Studies have shown healthy subjects have an average level of 30 ng/mL cell-free DNA, while cancer patients' levels are elevated to an average of 180 ng/mL, with some levels increasing to 5000 ng/mL.^76^ The system showed high selectivity for the survivin DNA sequence, with no response observed when the system was exposed to mismatched DNA sequences differing by one- or three-bases (Figure 4B). The system was then evaluated using the μPAD platforms and showed equally successful results. By analyzing the fluorescence in analysis zone 2, survivin concentrations in buffer, artificial saliva, artificial urine, and human serum led to increasing fluorescence signals (Figure 4C,D). The detection levels were similar but slightly higher on the μPAD, with the 5 nM data point showing an elevated, but not significant signal. The 50 nM (313 ng/mL) data point was well above the background and still in the range to detect elevated cell-free DNA concentrations in cancer patients. However, if lower detection limits are desired, the nanoparticle/μPAD detection system could be coupled with recombinase polymerase amplification (RPA). RPA is an isothermal alternative to PCR that works isothermally at 37 °C, meaning amplification would be possible by simply holding the tube in one’s hand or placing it in a warm area. Amplification could occur in as little as 10 min and not add to the complexity of the assay.^77^ This would increase the concentration of the target DNA to well above the demonstrated detection capabilities of the system. The μPAD also demonstrated the high selectivity shown in solution, with the one- and three-base mismatched DNA sequences resulting in no change in the fluorescence signal. While a micropipette was used to load 40 μL of sample solution into the μPAD, future iterations could include the use of a calibrated dropper to continue to improve ease-of-use.

**Figure 4:**
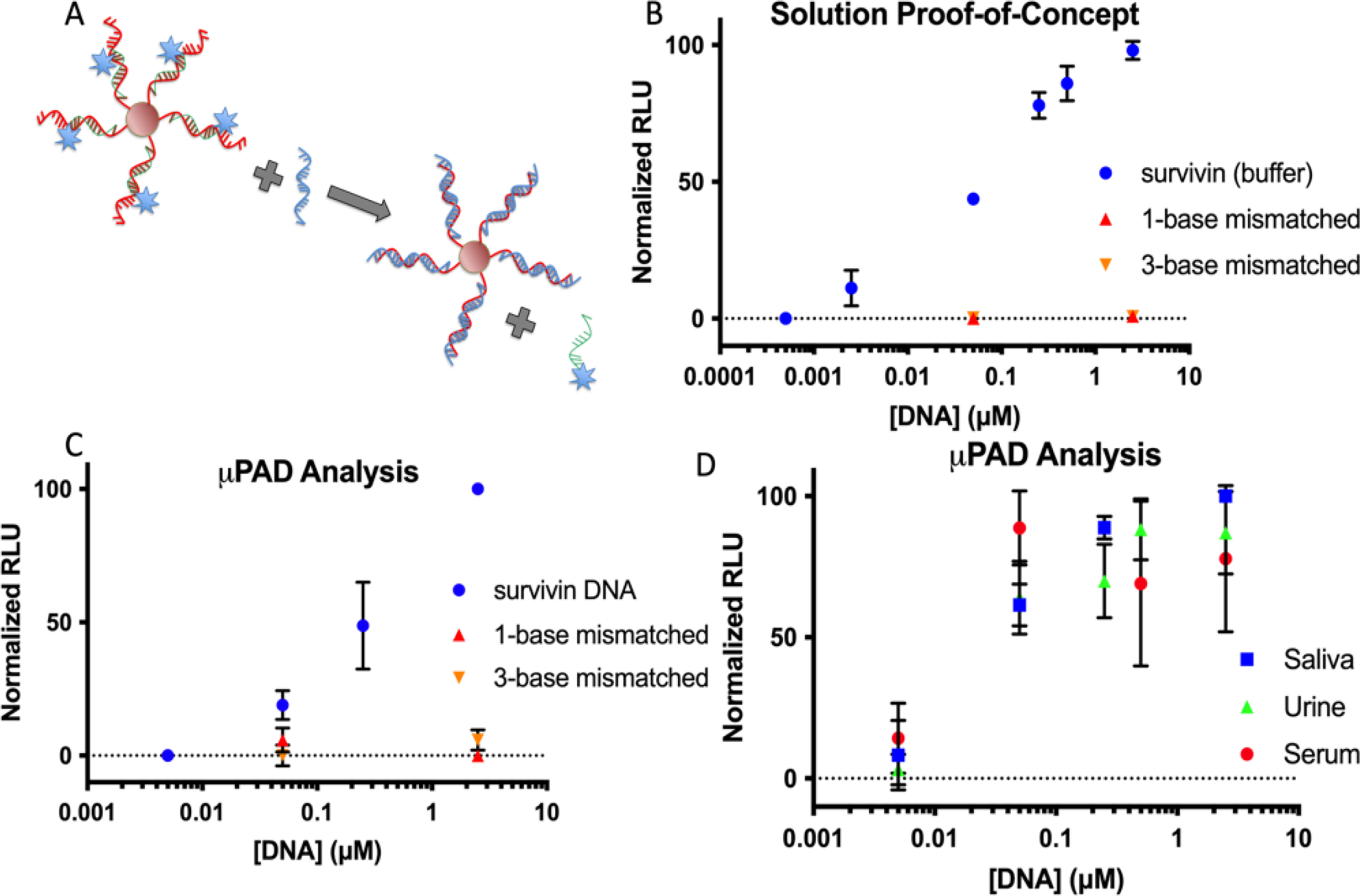
(A) Schematic of the release mechanism. (B) Solution based analysis of the detection system with fluorescence quenching. (C) μPAD detection of survivin DNA and one- or three-base mismatched sequences in buffer. (D) μPAD detection of survivin DNA in additional matrices.

Notably, while the proof-of-concept for the mechanism was demonstrated, further optimization of the nanoparticle system is still needed before it is clinically relevant. As shown in Figure 4D, different matrices impacted the assay performance. Serum is noticeably more viscous than urine or saliva, which affects the ability of the sample solution to wick down the paper-based platform. The ability of the flare to travel seamlessly to the detection zone affected the subsequent signal quantification, with variability seen in the larger associated uncertainty in serum. Dilution or extraction of the DNA from serum may aid in the reproducibility, as well as investigating additional platform materials for future iterations, as the move toward clinical relevance is pursued.

## Conclusions

Fe_3_O_4_@Au nanoparticles were engineered to selectively respond to increasing survivin DNA target concentrations that were quantified on a wax-printed μPAD platform. To our knowledge, Fe_3_O_4_@Au nanoparticles have not been implemented as the platform for analyte recognition and signal production with the proposed mechanism described herein or used in combination with μPAD technology to make POC devices. With regard to DNA/miRNA biomarkers, the demonstrated system fills major voids in the current lateral flow landscape. Specificity of DNA-based assays in the LFA format is lacking due to the requirement of high-stringency washing to remove nonspecific interactions.^78^ The demonstrated system eliminates the need to wash nonspecific interactions, as the flare is only released upon the specific interaction with the target DNA. The work could also find relevance with miRNA detection, with current miRNA detection proving extremely difficult due to the short sequence composition. With PCR, the reduced length of the molecule makes designing PCR primers challenging. The short length also makes detecting miRNA by an LFA platform problematic, as multiple probes are needed to bind the target miRNA to the test line and allow the detection mechanism to bind.^79^ Eliminating the reliance on antibodies and nitrocellulose membranes significantly reduced the cost to produce tests and will increase accessibility across the globe.

While the data suggest that the system is ready for translation of the mechanism and further development to a POC diagnostic system with biological fluids, there are still critical milestones to be addressed. Beyond continued optimization of the platform for all biological matrices, the detector portion of the sensing system will also need to be updated for use in remote applications. Here, a plate reader was used to quantify the fluorescence in the detection zone, which would be expensive and cumbersome for some situations in which POC diagnostics would be useful. As the move toward impact progresses, incorporation of a portable, potentially cellphone based readout mechanism, such as that described by Wang et al.,^80^ would be useful to for this analysis mechanism as well. Real samples would also need to be validated against PCR analysis to confirm the performance in clinical applications.

Further development for the implementation of the proposed system offers advantages over the currently available options and lays the foundation for an additional platform on which POC diagnostics can be built.

## Abbreviations

μPAD: microfluidic paper-based analytical device
Au: gold
Fe_3_O_4_@Au: gold-coated iron oxide nanoparticles
LFA: lateral-flow assay
POC: point-of-care
